# Fear of missing organisms (FOMO): Diabetic foot and osteomyelitis management opportunities

**DOI:** 10.1017/ash.2023.212

**Published:** 2023-09-29

**Authors:** Morgan Morelli, Andrea Son, Yanis Bitar, Michelle Hecker

## Abstract

**Background:** Hospitalizations for diabetic foot infections and lower-extremity osteomyelitis are common. Use of empiric antibiotic therapy for methicillin-resistant *Staphylococcus aureus* (MRSA) and *Pseudomonas aeruginosa* is also common. Guidelines recommend antibiotic therapy based on severity of illness, risk factors for MRSA and *P. aeruginosa*, and local prevalence. We evaluated the concordance between empiric antibiotic therapy and both culture results and definitive antibiotic therapy with a focus on MRSA and *P. aeruginosa*. We also evaluated how well MRSA and pseudomonal risk factors were predictive of culture results with these organisms. **Methods:** We conducted a cohort study of all patients admitted to our hospital system in 2021 with a diagnosis of a diabetic foot infection or lower-extremity osteomyelitis. Patients were included if they had an *International Classification of Disease, Tenth Revision* (ICD-10) diagnosis code of M86, E10.621, E11.621, or E08.621. Patients were excluded if antibiotics were for another indication or if they were aged <18 years. In patients with multiple hospitalizations only the first hospitalization was included. Empiric antibiotic therapy included antibiotics started by the admitting team. Definitive antibiotic therapy included the final antibiotic course either completed during admission or prescribed at the time of discharge. MRSA risk factors included prior positive culture with MRSA within the last year, hospitalization with IV antibiotics within 90 days, intravenous drug use, or hemodialysis. Pseudomonal risk factors included prior positive culture with *P. aeruginosa* within the last year or hospitalization with IV antibiotics within 90 days. **Results:** In 2021, 260 unique patients were admitted with suspected diabetic foot infections or lower-extremity osteomyelitis. 68 patients had >1 admission. Empiric anti-MRSA and antipseudomonal therapy was administered to 224 (86%) and 214 (82%) patients, respectively. Definitive anti-MRSA and antipseudomonal therapy was administered to 76 (30%) and 51 (20%) patients, respectively. Of the 195 patients who had wound cultures, 29 (15%) and 18 (9%) had positive cultures for MRSA and *P. aeruginosa* respectively (Fig.). The negative predictive value of MRSA risk factors for predicting a negative culture with MRSA was 91%. The negative predictive value of pseudomonal risk factors for predicting a negative culture with *P. aeruginosa* was 95%. **Conclusions:** Our data suggest an opportunity for substantial reductions in empiric anti-MRSA and antipseudomonal therapy for diabetic foot infection and lower-extremity osteomyelitis. The absence of MRSA and pseudomonal risk factors was reasonably good at predicting the absence of a positive culture with these organisms.

**Figure:**
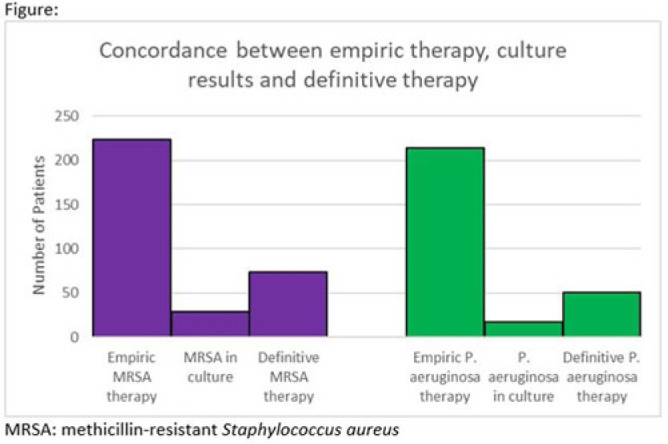

**Disclosure:** None

